# Emergence of input selective recurrent dynamics via information transfer maximization

**DOI:** 10.1038/s41598-024-64417-6

**Published:** 2024-06-13

**Authors:** Itsuki Kanemura, Katsunori Kitano

**Affiliations:** 1 Graduate School of Information Science and Engineering, Ritsumeikan University, 2-150, Iwakuracho, Ibaraki, Osaka 5670871 Japan; 2 Department of Information Science and Engineering, Ritsumeikan University, 2-150, Iwakuracho, Ibaraki, Osaka 5670871 Japan

**Keywords:** Information transfer, Multi-objective optimization, Vision system, Reservoir computing, Genetic algorithm, Network models, Computational models

## Abstract

Network structures of the brain have wiring patterns specialized for specific functions. These patterns are partially determined genetically or evolutionarily based on the type of task or stimulus. These wiring patterns are important in information processing; however, their organizational principles are not fully understood. This study frames the maximization of information transmission alongside the reduction of maintenance costs as a multi-objective optimization challenge, utilizing information theory and evolutionary computing algorithms with an emphasis on the visual system. The goal is to understand the underlying principles of circuit formation by exploring the patterns of wiring and information processing. The study demonstrates that efficient information transmission necessitates sparse circuits with internal modular structures featuring distinct wiring patterns. Significant trade-offs underscore the necessity of balance in wiring pattern development. The dynamics of effective circuits exhibit moderate flexibility in response to stimuli, in line with observations from prior visual system studies. Maximizing information transfer may allow for the self-organization of information processing functions similar to actual biological circuits, without being limited by modality. This study offers insights into neuroscience and the potential to improve reservoir computing performance.

## Introduction

Information processing within the brain is facilitated by neural cells whose responses can be approximated by binary responses to external stimuli. Through the formation of intricate networks, these neurons encode information via temporal sequences of zeros and ones, a process reflecting dynamic circuitry. Importantly, this circuitry is not arbitrary; it undergoes adaptive modifications in its structure to optimally respond to specific tasks or stimuli. The cerebral cortex in the human brain, which comprises approximately 180 functional regions^1^, plays a pivotal role in information processing. Functionally specialized wiring patterns are evident not only across the cerebral cortex but also within the microscopic receptive fields of sensory organs, indicating a widespread organizational principle. These patterns are not mere byproducts of postnatal learning but are instead genetically pre-determined through evolutionary processes, shaped by traits honed over millennia^2–7^. Despite the recognized importance of modular organization in neural processing, the mechanisms underlying^2,3,8^ the formation and refinement of these structures are not fully understood. This study aims to identify the principles guiding specialized wiring pattern formation in a visual system by integrating information theory with evolutionary computational algorithms.

Information theory, introduced by Claude Shannon^9,10^, has applications across diverse domains. It forms the basis for models, as shown by Linsker^11^, that capture the response characteristics of visual neurons through principles of information theory and Hebbian learning^12^. Further developments by Bell and Sejnowski^13^ have demonstrated that maximizing information transmission efficiency is key to modeling sensory systems. Although these applications have mainly focused on feedforward networks, the inclusion of feedback inputs from the cortex, as expanded upon by Tanaka et al., introduces the concept of recurrent infomax^14^, which is applicable to a variety of sensory systems^14–17^ and regarded as a core principle in sensory system architecture.

Moreover, the brain has been shown to exhibit wiring cost minimization^18^—represented by axon length and synaptic strength—which naturally limits long-distance connections and promotes the formation of modular, localized, and densely connected networks. Research by Ellefsen^19^, utilizing genetic algorithms in feedforward neural networks, has shown that reducing connection costs leads to the development of modules specialized for different tasks. This principle has been expanded to interconnected networks, further illustrating how minimizing connection costs and defining subtypes within networks enhance adaptability and performance across a range of tasks, as demonstrated by Yang et al.^20^, Kawai et al.^21^ and Tsuda^22,23^. They incorporated the task achievement (e.g., accuracy) calculated from supervised data as an objective function to let the system obtain an optimal solution to the task. In addition to structuring, supervised learning to minimize errors was also strongly optimized for the task. However, since the structure of the visual system in this study was acquired before birth, no supervised signal is considered to exist. In addition, wiring plasticity due to spontaneous firing occurs prenatally^24,25^, and learning occurs in a neural activity-dependent manner, such as defined by Hebbian rules, rather than in an error-minimizing manner.

This study aims to further contribute to the elucidation of the principles of wiring pattern formation from a mathematical model approach by searching for wiring patterns that maximize information transfer using genetic algorithms, clarifying their structure and internal information processing, and comparing the results with findings in actual biological systems. The results revealed that efficient information transfer needs sparse circuits with internal modular structures that use distinct wiring patterns. The stimuli selective responsiveness of the developed wiring pattern indicated functionalities akin to natural visual systems.

## Methods

### Circuit model

**Figure 1:**
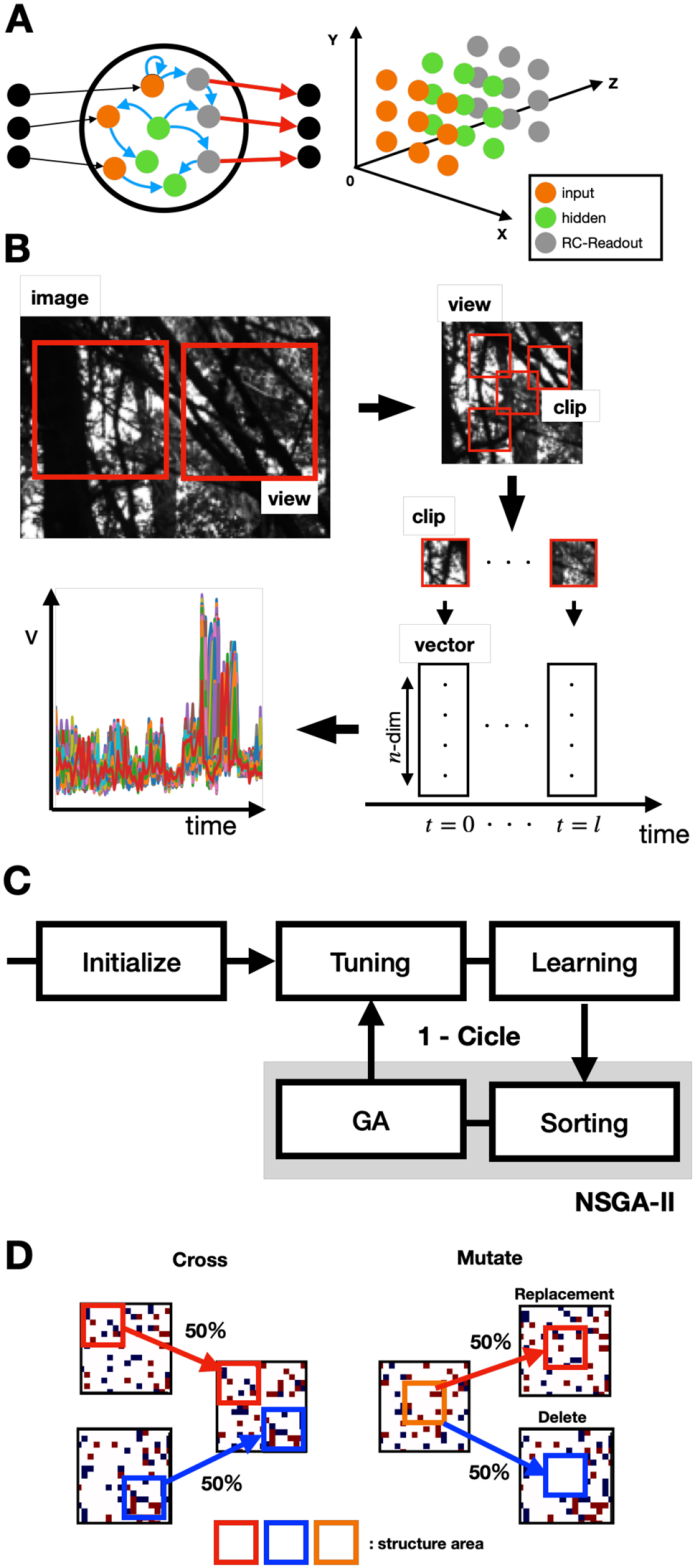
Experimental framework overview. (**A**) Circuit model and spatial arrangement. (Left) Depiction of the reservoir computing model illustrating the circuit framework. (Right) Spatial layout of reservoir units, highlighting their organized distribution. (**B**) Input signal generation process. Diagram detailing the methodology for generating input signals for the experiment. (**C**) NSGA-II experimental procedure. Flowchart depicting the non-dominated sorting genetic algorithm-II (NSGA-II) process applied in the experiment. (**D**) Crossover and mutation mechanisms. Left: Illustration of the crossover process with regions highlighted in red, blue, and orange indicating structural domains involved. Right: Depiction of the mutation process, showcasing the structural alterations within the specified domains.

In this study, we utilize evolutionary algorithms to enhance a comprehensive model of reservoir computing (RC), depicted in Fig. 1A (Left) as a recurrent neural network variant. Central to RC is the Echo State Network (ESN), a model introduced by Jaeger et al. in 2001^26,27^, which we equate with RC for the purposes of this study. The RC framework comprises three layers: an input layer, a reservoir layer, and a readout layer. Notably, plasticity is confined to the readout connections, with all other weights maintained at their initial settings. The dynamics within the reservoir layer are governed by an equation employing the hyperbolic tangent (tanh) activation function. In this context, bold uppercase letters denote matrices, while bold lowercase letters signify column vectors (${\varvec{x}}={({x}_{0},\dots , {x}_{n})}^{T}$). ${{\varvec{W}}}_{{\varvec{i}}{\varvec{j}}}$ denotes the element at the i-th row and j-th column of matrix ${\varvec{W}}$, and ${{\varvec{x}}}_{i}$ represents the i-th element of the column vector.

$${\varvec{x}}_{{\varvec{i}}} \left( {t + 1} \right) = {\varvec{x}}_{{\varvec{i}}} \left( t \right) + \alpha \user2{ }\mathop \sum \limits_{{\varvec{j}}} {\varvec{W}}_{{{\varvec{ij}}}} {\varvec{r}}_{{\varvec{j}}} \left( t \right) + \beta {\varvec{u}}_{{\varvec{i}}} \left( t \right) + \sigma \xi ,$$

where $${x}_{i}$$ represents the dynamics of the i-th unit in the reservoir, $${u}_{i}$$ represents the external input, $${r}_{i}=\text{tanh}({x}_{i})$$, $${\varvec{W}}$$ denotes the matrix of internal connectivity within the reservoir layer, and $$\xi$$ represents noise. $${\varvec{W}}$$ is the focus of the evolutionary algorithm, and at the time of initial pattern generation, the coupling strengths within $${\varvec{W}}$$ are generated from a uniform distribution of [−0.5, 0.5], establishing sparse connections where only 20% are nonzero. The spectral radius of $${\varvec{W}}$$ is adjusted to unity. Constants $$\alpha$$, $$\beta$$, and $$\sigma$$ represent the decay coefficient and strength coefficients, respectively. The model's output ($${z}_{i})$$ is calculated as follows:

$$z_{i} \left( {t + 1} \right) = \mathop \sum \limits_{j} {\varvec{W}}_{ij}^{out} \widehat{{x_{j} }},$$

where $\widehat{{\text{x}}_{\text{j}}}$ represents the dynamics of reservoir units connected to the readout (RC-Readout units), and ${\mathbf{W}}_{\text{ij}}^{\text{out}}$ is the matrix of coupling strengths for the readout connections, initially set to a normal distribution with a mean of zero and variance of one. The RC model employed in this study incorporates three distinct unit subtypes within the reservoir layer: 1) input units that receive external inputs, depicted in Fig. 1A as orange units; 2) RC-readout units that have connections to the readout layer, shown as gray units; and 3) hidden units, which encompass the remaining units, illustrated as green units. Consequently, no units simultaneously receive input and connect to the readout layer. The circuit model parameters were set with a total of 48 units across the RC model, distributed evenly with 16 units per subtype, including the readout units. The internal connection sparsity was established at $s=0.2$ (20%), with $\alpha$, $\beta$, and $\sigma$ parameters configured to $\alpha =0.1$, $\beta =1.0$, and $\sigma =0.01$, respectively. The noise $\xi$ follows a normal distribution with a mean of 0 and a variance of 1.

### Input signal

For input signal construction, we utilized time-series data derived from grayscale images of natural landscapes, sourced from the Van Hateren natural image database. The generation process, illustrated in Fig. 1B, began with extracting multiple scene images of arbitrary sizes from each landscape image. By randomly shifting smaller square regions within each scene image in various directions, we produced multiple clipped images. These clipped images were then linearized into vectors, with each vector element representing a scaled pixel value within [0, 1]. This sequence of linearized images formed the time-series input, with each stimulus presented in a singular time step. The approach mimics saccadic eye movements to simulate real eye movements closely. In our experiments, five landscape images were used for training and two for validation. From each landscape image, 100 scene images were extracted, yielding 40 clipped images per scene, with the clipped images standardized to a size of 4. This methodology not only utilizes natural visual stimuli but also incorporates the dynamic nature of visual processing through the simulation of saccadic movements.

### Non-dominated sorting genetic algorithms-II (NSGA-II)

The non-dominated sorting genetic algorithm-II (NSGA-II)^28^ is an evolutionary computation technique tailored for multi-objective optimization challenges where the presence of competing objectives necessitates the identification of a set of optimal solutions rather than a singular optimal outcome. NSGA-II facilitates the concurrent optimization of multiple objectives by employing non-dominated sorting and ranking strategies, enabling the formulation of a balanced solution set. This algorithmic approach maintains the general workflow of traditional genetic algorithms but is specifically enhanced for handling multi-objective problems.

In this study, we conceptualize the evolution of circuit structures within biological systems as a multi-objective optimization problem, hypothesizing that circuitry is optimized not solely for enhanced information transfer but also for minimized maintenance costs. Consequently, our optimization goals are twofold: to maximize information transfer efficiency and to minimize circuit maintenance expenses, such as metabolic costs. This allows for the exploration of wiring patterns (${\varvec{W}}$) that excel in information transfer while incurring low costs for circuit maintenance. For this purpose, we utilize transfer entropy to quantify information transfer and assess maintenance costs through metrics like coupling costs and network density.

### Procedure of NSGA-II experiment

Our experimental methodology, incorporating NSGA-II, is detailed in Fig. 1C. The process begins with the initial generation of wiring patterns, followed by the adjustment of the spectral radius of $W$ to 1. Subsequently, the reservoir layer units undergo spontaneous firing for a designated period, during which internal connections are refined via Hebbian learning—a process inspired by retinal waves observed in the structural development of the retina^24,25,29–32^. Learning of the readout connections also proceeds through Hebbian learning, employing a training signal. Upon the completion of learning phases, evaluative metrics such as coupling cost, network density, and information entropy are computed. These metrics then guide the selection of surviving patterns and the generation of subsequent patterns through NSGA-II’s mutation and crossover mechanisms. This iterative cycle of tuning and generation is repeated for a predefined number of cycles, set in this study to $Gen=100,$ with each cycle comprising $N=P+Q=500$ patterns (where $P=250$ is preserved from the preceding generation and $Q=250$ is newly generated). Overall, the analysis incorporates a total of 25,250 patterns, with the tuning duration fixed at 100 timesteps.

### Generation of new wiring patterns in NSGA-II

The methodologies for crossover and mutation in generating new wiring patterns within the framework of NSGA-II are depicted in Fig. 1D. For pattern generation, inheritance, replacement, or omission of adjacent and diagonal regions surrounding any given point is incorporated. Crossover is executed by randomly selecting two parent patterns from the current generation’s population, with structural regions from each parent being inherited at a 50% ratio. Additionally, a subset of patterns in each generation is subjected to mutations with an 80% ratio. During mutations, structural regions are either completely removed (rendered as zeros) with a 50% probability, or new structural regions are introduced for the remaining 50% of mutations, drawing from a uniform distribution within [−0.5, 0.5] and adhering to the initial sparsity rate of connections. The structural regions considered for these operations are defined as square areas with dimensions of 2 × 2.

### Hebbian rule

The Hebbian rule, named after Canadian psychologist Donald Hebb, who introduced it in 1949^12^, serves as a cornerstone of synaptic plasticity. It posits that the synaptic strength between two sequentially activated neurons is increased, encapsulating the principle “neurons that fire together, wire together.” In this study, we applied a subtractive normalized version of the Hebbian rule for synaptic adjustment, relevant both to the tuning of the reservoir layer and the training of readout connections. This variant of the Hebbian rule^12^ is mathematically represented as follows:

$$\tau \frac{{d{\varvec{w}}}}{dt} = v{\varvec{u}} - \frac{{v\left( {{\varvec{n}} \cdot {\varvec{u}}} \right){\varvec{n}}}}{N},$$

where $v$ represents the activity of the downstream unit, ${\varvec{u}}$ denotes the activity of the upstream unit, ${\varvec{n}}$ is an $N$-dimensional vector with all elements set to 1, and $N$ denotes the total count of upstream units. This modification subtracts the average activity product $v{\varvec{u}}$ from the conventional Hebbian learning rule, thereby normalizing synaptic adjustments. For the purposes of this study, $\tau$ was configured to 0.1. When tuning the reservoir layer, the total number of units in the reservoir layer, N, is 48, while for the learning of readout connections, N corresponds to the number of RC-Readout units, which is 16.

### Model’s coupling cost

In this study, we introduce and utilize the coupling cost as a critical evaluation metric to account for hardware constraints on the network structure. Hardware constraints are defined by the structural limitations imposed by spatial relationships and distances between units within the network. Reflecting the biological premise that neurons in closer spatial proximity are more likely to form connections than those further apart, we incorporate this spatial consideration into our pattern generation process.

The methodology for calculating the coupling cost is as follows: Initially, coordinates representing spatial location information in three-dimensional (3D) space are assigned to all units within the reservoir layer. The spatial arrangement of these units adopts a grid-like layered structure, as illustrated in Fig. 1A (right), with a uniform distance of 1 between adjacent units. The coupling cost (${c}_{ij}$) between any two units ($i$ and $j$) is determined by the formula:

$$c_{ij} = {\varvec{W}}_{ij} {\varvec{D}}_{ij} .$$

where ${\varvec{W}}$ represents the matrix of internal coupling strengths within the reservoir layer, where ${{\varvec{W}}}_{ij}$ is the specific coupling strength between units $i$ and $j$, and ${{\varvec{D}}}_{ij}$ represents the shortest physical distance between these units. The shortest distance is calculated using the Warshall-Floyd algorithm. The overall coupling cost ($C$) for the network is then defined as the cumulative sum of all individual c_ij_ values across the network, expressed as:

$$C = \mathop \sum \limits_{i,j} c_{ij} .$$

### Model’s density

Furthermore, this experiment employs network density as a proxy for the maintenance or energy cost associated with sustaining the network’s functionality. Maintenance costs refer to the energetic expenditure required to preserve the network’s structural integrity. Given that higher-density network structures inherently demand more energy for maintenance due to increased connection counts, network density serves as a significant constraint in our structural generation, aiming to balance efficient information processing with sustainable energy consumption. This approach aligns with the understanding that in neural circuits, metabolic considerations necessitate energy to maintain connections.

### Model’s modularity

To assess the modularity of circuit structures in this study, we employed Newman modularity^33,34^, a measure that quantifies the quality of a network's division into modules or communities. High modularity indicates a network structure where connections are densely packed within modules while being sparse across different modules, reflecting a high degree of structural organization within the functional circuitry. Modularity is calculated as the difference between the actual fraction of connections within modules and the expected fraction in a hypothetical random network, emphasizing the network’s division efficiency into distinct functional groups. We determined modularity by identifying the partitioning pattern that yields the highest modularity value and allocating units to modules accordingly, using this as a metric for evaluating network structure.

### Transfer entropy

Transfer Entropy (TE)^35^ serves as a tool in information theory to quantify the causal influence one time series has on another, distinguishing it from mutual information by its ability to detect changes in mutual dependence over time. This metric is particularly relevant in neuroscience, where stimuli often precede brain activity, suggesting a directional flow of information crucial for brain function. In our study, we opted for TE over mutual information to incorporate the aspect of causality into our analysis of brain circuit development. It can be expressed as:

$$TE_{X \to Y} = P\left( {Y_{t + 1} ,Y_{t}^{\left( k \right)} ,X_{t}^{\left( l \right)} } \right)\log \left( {\frac{{P\left( {Y_{t + 1} |Y_{t}^{\left( k \right)} ,X_{t}^{\left( l \right)} } \right)}}{{P\left( {Y_{t + 1} |Y_{t}^{\left( k \right)} } \right)}}} \right).$$

The formula for TE assesses the impact of past states of two variables (${Y}_{t}^{(k)}$ and ${X}_{t}^{(l)}$) on the future state of ${Y}_{t+1}$, with a TE value of zero indicating no influence from $X$ to $Y$ and higher values signifying stronger causal relationships. This measurement is instrumental in understanding the directional flow of information in the network, especially in examining how the dynamics within the input signal (X) influence the readout unit's dynamics (Y), offering insights into the developmental mechanisms of brain circuitry.

### Mutual information

Mutual Information (MI) quantifies the statistical dependency between two random variables, X and Y, serving as a measure of the shared information that X provides about Y and vice versa. This metric, however, is static and does not account for the temporal dynamics or causal relationships that might exist between variables. MI, $I\left(X;Y\right),$ is calculated using a specific formula that evaluates the shared entropy between X and Y, reflecting the level of informational overlap as

$$I\left( {X;Y} \right) = \mathop \sum \limits_{{\left\{ {x \in X} \right\}}} \mathop \sum \limits_{{\left\{ {y \in Y} \right\}}} p\left( {x, y} \right){\text{log}}\left( {\frac{{p\left( {x, y} \right)}}{p\left( x \right)p\left( y \right)}} \right).$$

In our study, MI was utilized to quantitatively analyze the system dynamics by computing the MI of trajectories derived from time-series data. This approach facilitates the examination of dependencies between variables at discrete time points and the overall transfer of information within the system. MI thereby emerges as a crucial tool for elucidating the interactions between variables in the system and deciphering the dynamics’ underlying characteristics.

### Evaluation of separation degree in the spatial domain of trajectories

For a more refined analysis, this study utilizes normalized MI (NMI) to gauge the separability in the spatial domain of trajectories, functioning as an indicator of classification precision in trajectory categorization. A high degree of separation indicates clear classification of trajectories in response to different stimuli, correlating with improved classification accuracy for each stimulus type. During each stimulus presentation, trajectories were classified spatially using the k-means clustering method, and labels were assigned accordingly. The separation degree was then quantified by calculating the NMI between the actual stimulus labels and those derived from k-means clustering, using the formula:

$$NMI\left( {C, K} \right) = \frac{{I\left( {C;K} \right)}}{{\sqrt {\left\{ {H\left( C \right)H\left( K \right)} \right\}} }},$$

where NMI is normalized within the range [0,1], where higher values denote greater classification accuracy and clearer trajectory segregation. Here, *C* and *K* denote the true labels associated with stimulus presentations and the labels acquired through k-means classification, respectively. The entropy $H(\cdot )$ is defined by:

$$H\left( {\varvec{C}} \right) = - \mathop \sum \limits_{{\left\{ {i = 1} \right\}}}^{k} P\left( {C_{i} } \right)log\left( {P\left( {C_{i} } \right)} \right).$$

where k is the number of classes and $P({C}_{i})$ represents the proportion of class ${C}_{i}$ in the dataset.

### Fractal dimension

Fractal dimension (FD) serves as a quantitative measure for evaluating the self-similarity and complexity of an object. In our study, we estimated the FDs of trajectories using the box-counting method. This method entails overlaying the object of interest with a grid of boxes, each having a side length of $\epsilon ,$ and counting the number of points of the object, *N(ϵ)*, contained within each box. This counting process is iterated with varying box sizes. The FD is then determined using the formula:

$$FD= \underset{\{\epsilon \to 0\}}{\text{lim}}\frac{\text{log}(N(\epsilon ))}{\text{log}(1/\epsilon )}.$$

This formula evaluates the space that an object fills as the box size approaches infinitesimally small values. An object’s complexity increases with higher FD values, indicating a more intricate fractal structure. In our analysis, we explored the FDs of trajectories within a low-dimensional space to evaluate their complexity and stability, providing insights into the spatial dynamics and structural intricacies of the system under study.

### Trajectory volume

Furthermore, to assess the spread of trajectories within a 3D space, we introduced the concept of trajectory volume. The position of a trajectory at any given time t is represented by $f(t)=[x(t),y(t),z(t)]$. The trajectory volume, encapsulating the overall spread of these trajectories in 3D space, is defined by the following formula:

$$V = \iiint_{{\text{D}}} f\left( t \right)dx\, dy\, dz\, dt.$$

The volume is normalized to a maximum value of 1 to facilitate comparison.

## Results

### Wiring modification experiment results

**Figure 2:**
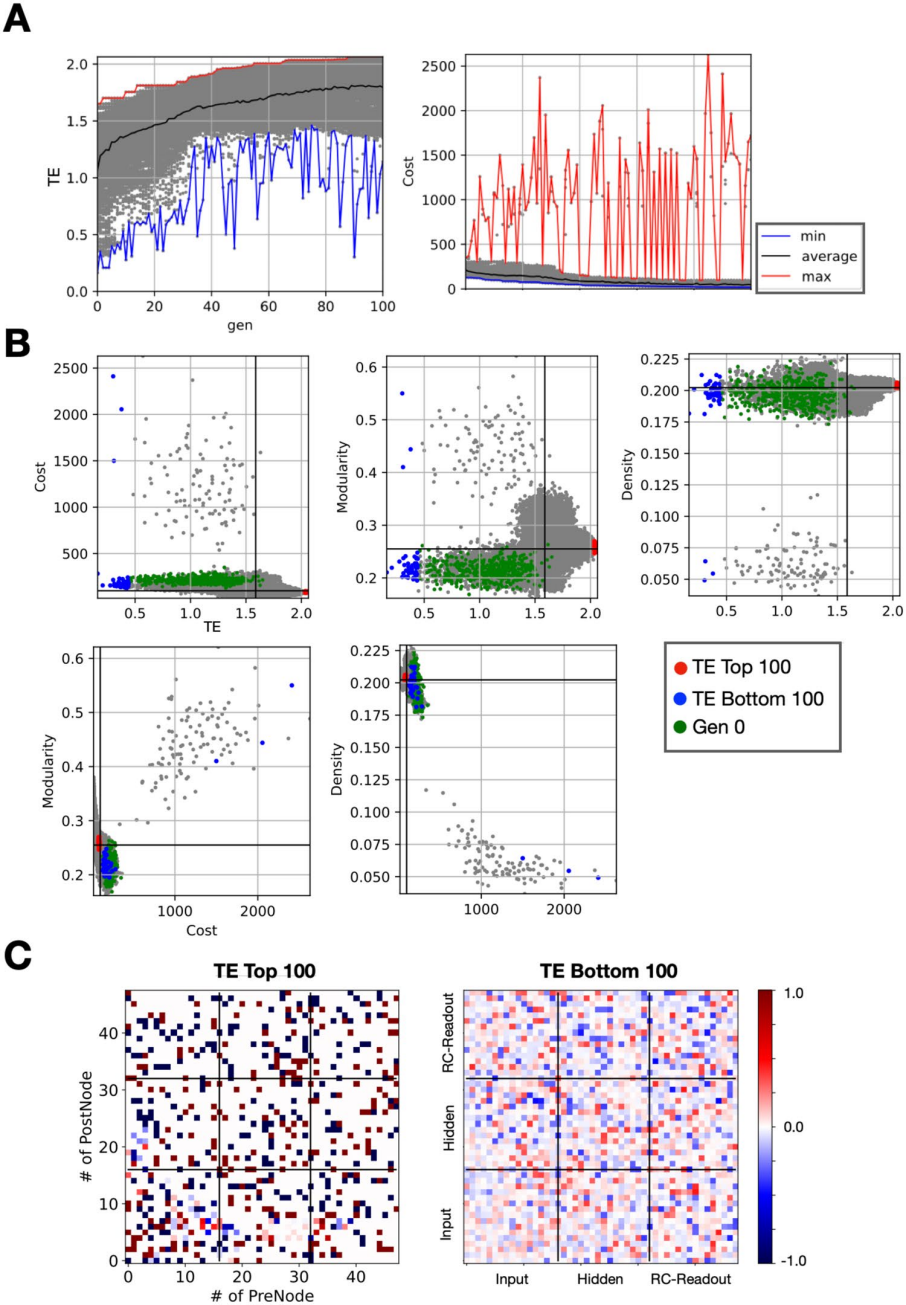
Information maximization and connection cost minimization outcomes. (**A**) Transition of evaluation metrics across generations. Graphs showing the progression of maximum (red line), minimum (blue line), and average (black line) values of each evaluation metric over generations, with gray points marking individual pattern entities. (**B**) Evaluation metric distributions. The scatter plot shows individual pattern entities (gray points) and categorizes them based on transfer entropy (TE) performance: top 100 (red points), bottom 100 (blue points), and initial generation (green points). Solid black lines indicate average values for each metric. (**C**) Structural connectivity among the top 100 entities (Left) and among the bottom 100 entities (Right). Representation of connection patterns with the horizontal axis denoting the transmitting side (pre-side) unit number and the vertical axis for the receiving side (post-side) unit number. Excitatory connections are marked in varying intensities of red, while inhibitory connections are in blue. The color intensities represent the rates of connections. Black lines delineate unit subsets (see methods section for details).

In Fig. 2, the experimental outcomes are depicted, focusing on the objectives of increasing transfer entropy (TE) and diminishing coupling costs as pivotal objective functions. TE is an index that quantifies the amount of information transferred while considering causal relationships. The higher the TE of a wiring pattern, the more efficient the information transfer from the input unit to the RC readout unit. Figure 2A depicts the generational changes in the evaluation values for the surviving wiring patterns (${\varvec{W}}$) across successive generations. This progression demonstrates an upward trend in TE alongside a reduction in coupling costs, indicating the effective evolution towards the intended pattern characteristics. Specifically, the red, blue, and black lines illustrate the maximum, minimum, and average evaluation values, respectively, across generations, with gray dots representing individual pattern entities. Notably, while the initial generation’s TE was approximately 1.0, it experienced a substantial increase to approximately 2.0, doubling through successive modifications. Concurrently, the coupling cost, which represents the cost of coupling between units, was reduced from approximately 250 to approximately 100.

Figure 2B presents the distribution of evaluation metrics for each pattern, including TE, coupling costs, modularity, and network density. Here, gray dots mark the positions of individual patterns, with red and blue dots highlighting the top 100 patterns for TE (${{\varvec{W}}}_{{\varvec{h}}{\varvec{i}}{\varvec{g}}{\varvec{h}}}$) and the bottom 100 for TE (${{\varvec{W}}}_{{\varvec{l}}{\varvec{o}}{\varvec{w}}}$), respectively, and green dots representing the 500 initial generation patterns (${{\varvec{W}}}_{{\varvec{i}}{\varvec{n}}{\varvec{i}}{\varvec{t}}}$). Solid black lines denote the average values for each evaluated metric.

The analysis from Fig. 2B reveals that ${{\varvec{W}}}_{{\varvec{h}}{\varvec{i}}{\varvec{g}}{\varvec{h}}}$ generally features marginally lower coupling costs compared to the average, with a slight uptick in modularity. However, given the inherent relationship between reduced coupling costs and increased modularity, the emergence of modular structures cannot be conclusively determined from this result alone. The density metric remained relatively stable at approximately 0.2 across all evaluations.

Despite the constant density, the observation of reduced coupling costs and heightened modularity in ${{\varvec{W}}}_{{\varvec{h}}{\varvec{i}}{\varvec{g}}{\varvec{h}}}$ implies that the wiring patterns underwent modifications, possibly due to alterations in connection strengths and the spatial arrangement of interconnected units. These changes hint at the potential development of some degree of modular structure within the wiring patterns. The bottom section of Fig. 2B, illustrating the interplay between coupling costs, modularity, and density, further corroborates the existence of correlations among these evaluation metrics.

Figure 2C provides a visual comparison between the wiring patterns of ${{\varvec{W}}}_{{\varvec{h}}{\varvec{i}}{\varvec{g}}{\varvec{h}}}$ (left) and ${{\varvec{W}}}_{{\varvec{l}}{\varvec{o}}{\varvec{w}}}$ (right) within the reservoir layer, specifically highlighting the excitatory and inhibitory connections. The horizontal axis denotes the unit number on the information-sending side (Pre side), and the vertical axis denotes the unit number on the receiving side (Post side), with red and blue representing the rates of excitatory and inhibitory connections, respectively. Dark red color (closer to +1) indicates a greater number of entities with excitatory connections, while dark blue color (closer to −1) indicates a greater number of entities with inhibitory connections. Black lines demarcate subsets within the units. While ${{\varvec{W}}}_{{\varvec{h}}{\varvec{i}}{\varvec{g}}{\varvec{h}}}$ displays discernible optimal structures, ${{\varvec{W}}}_{{\varvec{l}}{\varvec{o}}{\varvec{w}}}$ lacks clear patterns. Analysis of the average clustering coefficient and the average shortest path length, both yielding values of approximately 0.2 and 2.0, respectively, for ${{\varvec{W}}}_{{\varvec{h}}{\varvec{i}}{\varvec{g}}{\varvec{h}}}$ and ${{\varvec{W}}}_{{\varvec{l}}{\varvec{o}}{\varvec{w}}}$, does not conclusively indicate the presence of small-world network properties, a structure characterized by high clustering and short path lengths^36–39^.

### Differences in wiring modifications due to objective functions

In the broader context of Fig. 2, the experimental outcomes underscore the effects of prioritizing an increase in TE and a reduction in coupling costs as core objective functions. This segment elaborates on a comparative analysis involving seven distinct combinations of objective functions across various experimental setups, each designed to explore different aspects of network wiring modifications:

- 1. Baseline condition (TE increase and coupling cost decrease): This scenario, represented in Fig. 2, serves as the foundational comparison point, focusing on enhancing TE while reducing coupling costs.
- 2. TE increase: Exclusively aims at augmenting the TE to understand its isolated impact.
- 3. Coupling cost decrease: Concentrates solely on minimizing the coupling costs, providing insight into its standalone effects.
- 4. Density decrease: Targets a reduction in network density, examining its influence on network structure and functionality.
- 5. Combined objectives (TE increase, coupling cost decrease, and density decrease): Integrates all three objectives to assess their synergistic effects on network optimization.
- 6. Dual objectives (TE increase and density decrease): Investigates the combined impact of increasing TE and decreasing network density.
- 7. Dual objectives (coupling cost decrease and density decrease): Explores the effects of simultaneously reducing coupling costs and network density.

An increase or decrease in each of the three objective functions (TE, coupling cost, and density) implies the following: TE is an index that quantifies the amount of information transferred considering causal relationships, with larger values indicating higher transfer efficiency.

Coupling cost is the cost of coupling between units. In actual neural circuits, coupling between physically distant neurons (high coupling cost) is suppressed. Low coupling costs indicate that units in the circuit are coupled to nearby units. Density represents the wiring density of a circuit, and in a neural circuit, circuits with more coupling between neurons (higher density) are considered to have higher maintenance costs. Circuits with low density indicate low maintenance costs.

**Figure 3:**
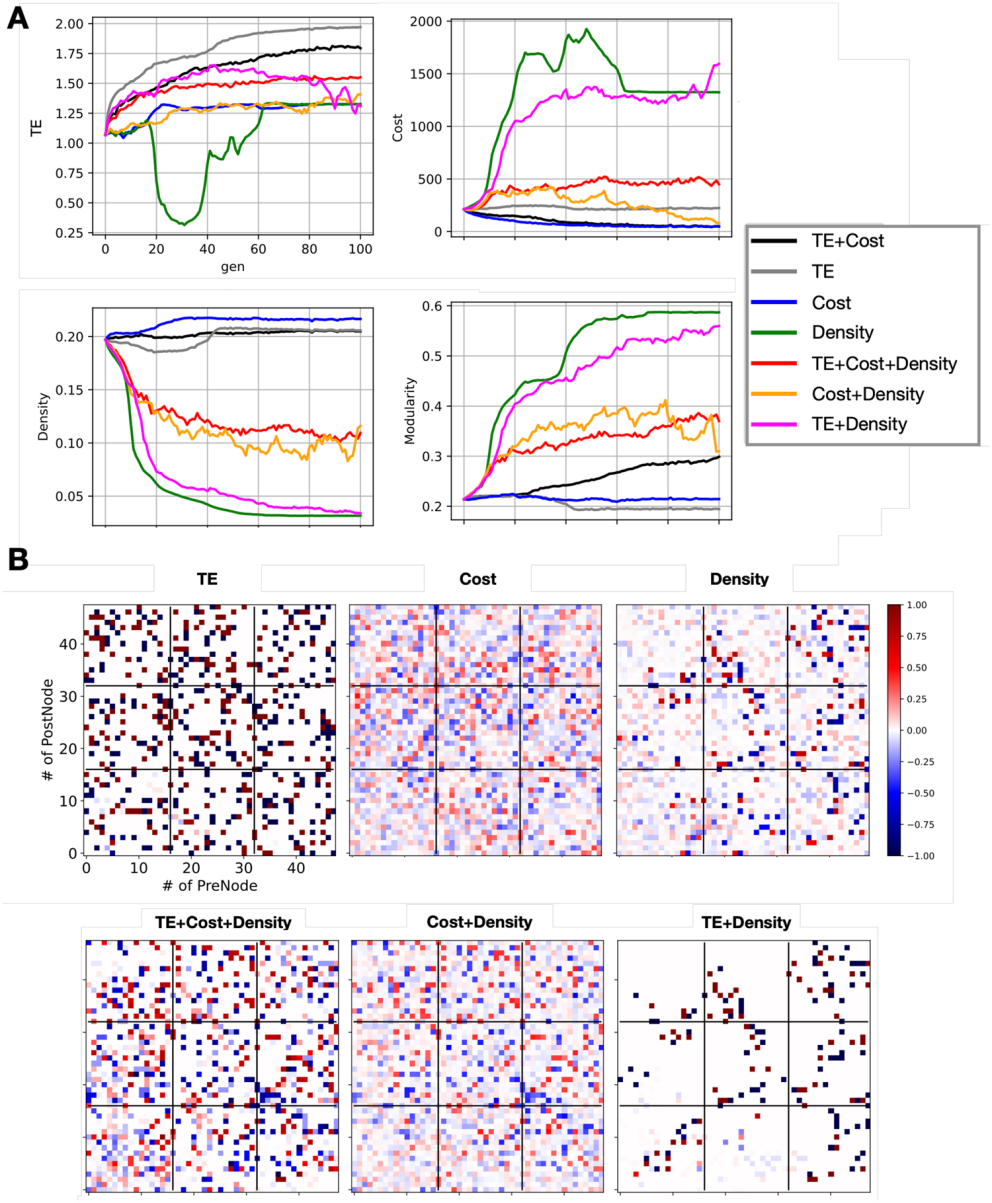
Impact of varying objective functions on generational development. (**A**) Transition of evaluation metrics across generations. The black line depicts the baseline scenario with objectives of increasing TE and decreasing connection cost. The grey line is for the scenario focusing solely on increasing TE, the blue line for solely decreasing connection cost, the green line for solely decreasing density, the red line for the combined objectives of increasing TE, decreasing connection cost, and decreasing density, the orange line for decreasing connection cost with an additional focus on decreasing density, and the pink line for increasing TE with a focus on decreasing density. (**B**) Structural connectivity among the top 100 entities for each condition. Stronger red (closer to +1) indicates a greater number of entities with excitatory connections, and stronger blue (closer to −1) indicates a greater number of entities with inhibitory connections. The upper row illustrates structural trends when focusing individually on increasing TE, decreasing connection cost, and decreasing density (from left to right). The lower row presents structural trends for combined objectives: increasing TE and decreasing connection cost, decreasing density, decreasing connection cost alongside decreasing density, and increasing TE with decreasing density (from left to right).

The results depicted in Fig. 3 provide a comprehensive overview of the effects of different objective function constraints on the generational evolution of wiring patterns within a neural network model. Figure 3A highlights the generational changes in the average values of TE, coupling cost, and network density under each experimental condition.

When the objective was solely to increase TE (represented by the gray line), the model generated wiring patterns with a high average TE, as expected. However, the associated coupling cost was higher compared to the baseline condition (TE increase + coupling cost decrease; condition 1), underscoring the trade-off between enhancing TE and managing coupling costs. The constant density metric (lower left of Fig. 3A) indicates that the observed pattern changes stem from alterations in the connectivity pairs and coupling strengths rather than from an increase in the number of connections. Notably, the modularity did not show an increase under this condition (lower right of Fig. 3A), suggesting that the constraint on TE alone did not lead to the formation of a modular structure when compared to the baseline condition.

Conditions that did not prioritize TE (Supplementary Materials) enhancement, focusing instead on combinations of coupling cost and density reductions (depicted by blue, green, and orange lines), did not demonstrate a significant increase in TE. This observation hints at the existence of inherent wiring configurations that are optimized for maximizing TE.

Figure 3B, similar to Fig. 2C (left), breaks down these findings by each experimental condition. The preservation of certain patterns, especially under constraints solely focusing on density reduction (upper-right), and the inclusion of TE as an objective (lower-right), suggests that these specific configurations are crucial for maximizing information transfer efficiency while also managing network density. The initial decrease in TE observed in conditions focused on density reduction (green line) points to the immediate impact of the objective function. However, the slight increase in TE at approximately Gen = 60 relative to the starting generation indicates that patterns optimized for reduced density can also inadvertently enhance information transfer efficiency. These insights reveal the complex interplay between various network characteristics and their collective impact on the functional optimization of neural network models, suggesting potential strategies for designing efficient and effective neural architectures.

### Network wiring pattern analysis

**Figure 4:**
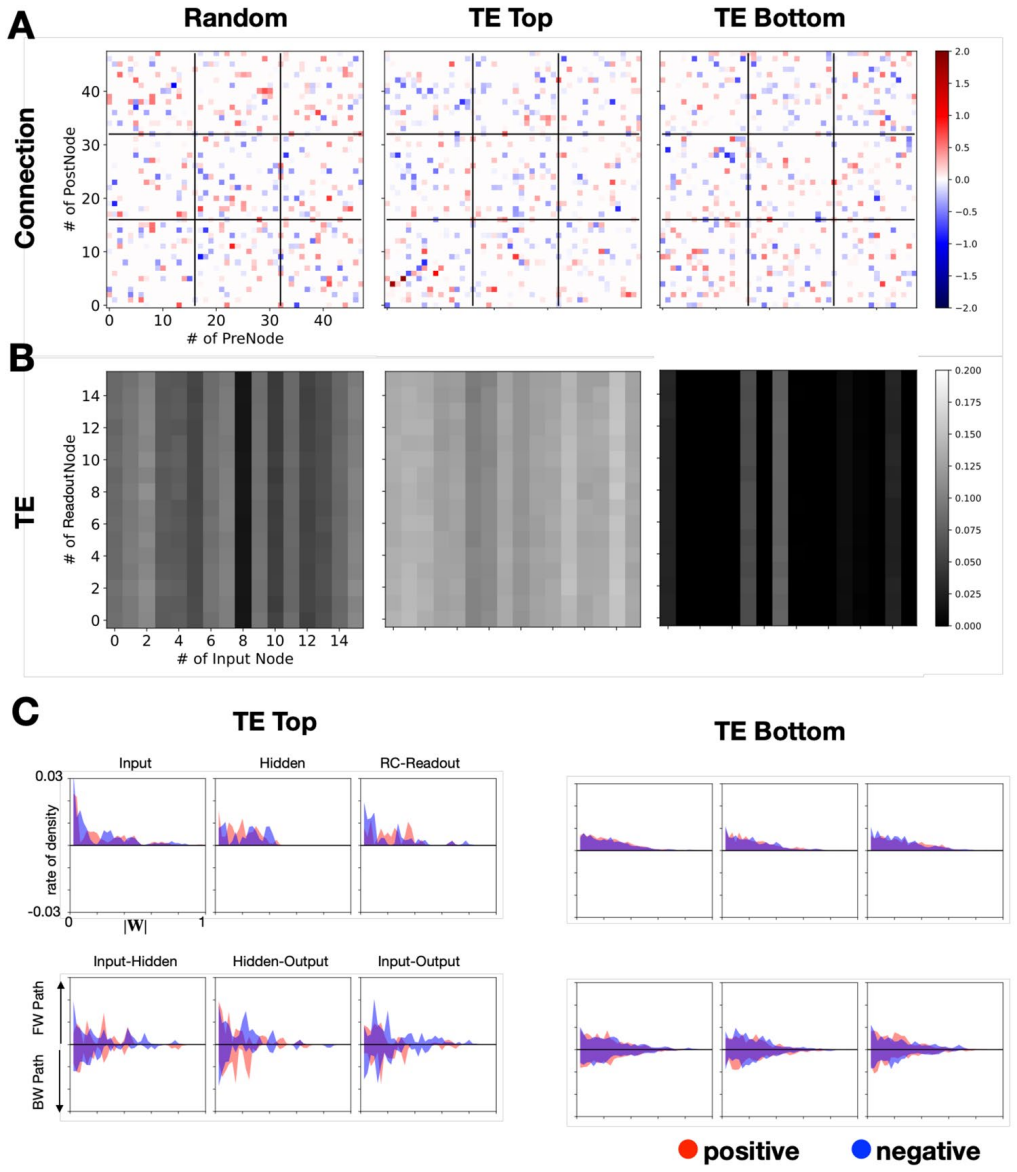
Network wiring pattern analysis results. (**A**) Connection strengths in the reservoir layer for three types of entities: those with random initialization, those with the highest TE, and those with the lowest TE. (**B**) Visualization of information transfer (TE) from input units to readout units for each type of entity. The axes denote the unit numbers for input (horizontal) and readout (vertical), with grayscale intensity indicating the TE amount. The sequence is from left to right: a randomly initialized entity, the entity with the highest TE, and the entity with the lowest TE. (**C**) Strength distribution of connections within and between unit subtypes in the reservoir layer. The upper row displays distributions within each unit subtype (input, hidden, and RC-Readout units, from left to right). The lower row illustrates distributions between subtypes (input-hidden, hidden-RC-Readout, and input-RC-Readout units), with a central horizontal line distinguishing the forward path (upper side) from the backward path (lower side). Excitatory connections are marked in red, inhibitory connections in blue, with axes showing connection strength (|W|) and density ratio.

The detailed analysis of wiring patterns within the reservoir layer, focusing on the basic conditions of increasing TE and reducing connection costs, reveals the intricate dynamics of internal connections. Figure 4A compares the pattern of random initialization of the pattern after wiring modifications: a randomly initialized pattern (${{\varvec{W}}}_{rand}$) (left), the pattern achieving the highest TE (${{\varvec{W}}}_{top}$) (center), and the pattern with the lowest TE (${{\varvec{W}}}_{bot}$) (right). This visual analysis indicates a lack of distinct wiring patterns or substantial changes in coupling density, suggesting that the variations in evaluation values are attributable to adjustments in connection pairs and their strengths rather than to a notable shift towards modularization or significant density alterations.

In Fig. 4B, the dynamics of information transfer from input units to readout units are graphically depicted, correlating with the configurations presented in Fig. 4A. The figure employs shading to represent the magnitude of TE, with the axes denoting the respective unit numbers. The ${{\varvec{W}}}_{rand}$ exhibits a skewed distribution of information transfer, peaking at a TE value of approximately 0.1, indicating a relatively low efficiency in information propagation. Conversely, ${{\varvec{W}}}_{top}$ demonstrates a uniform and elevated TE value of approximately 0.175 across all connections, signifying a robust and consistent information transfer mechanism. On the other hand, ${{\varvec{W}}}_{bot}$ is characterized by a conspicuous absence of effective information transfer, aligning with expectations that diversified readout unit activity correlates with increased TE.

Figure 4C delves into the density distributions of excitatory and inhibitory connection strengths within ${{\varvec{W}}}_{top}$ and ${{\varvec{W}}}_{bot}$, providing a detailed view of the internal connection dynamics in the reservoir layer. The figure is divided into two main sections: the upper row presents the distribution of connections within each subtype of the reservoir layer (input, hidden, and RC-Readout units), while the lower part focuses on the distribution between different subtypes (input-hidden, hidden-RC-Readout, and input-RC-Readout units). The central horizontal line serves as a reference point, with the upper part indicating the forward path from input to output and the lower part indicating the backward path from output to input. The distributions are marked by red for excitatory and blue for inhibitory connections, with connection strength (|W|) on the horizontal axis and the density ratio of each connection strength on the vertical axis.

A distinct difference in the distribution patterns between ${{\varvec{W}}}_{top}$ and ${{\varvec{W}}}_{bot}$ is observed, with ${{\varvec{W}}}_{bot}$ showing a more symmetrical distribution across the excitatory-inhibitory ratio as well as the forward and backward Paths. Conversely, ${{\varvec{W}}}_{top}$ exhibits a noticeable bias, indicating a divergence in the distribution of connection strengths.

These observations are consistent across the various experimental conditions outlined in Fig. 3, where ${{\varvec{W}}}_{top}$ is contrasted against ${{\varvec{W}}}_{bot}$. The analysis reveals that, except in scenarios focusing exclusively on density reduction or the combined objective of enhancing TE while reducing density, structural differences remain largely unchanged. The conditions prioritizing density adjustments or integrating both TE and density objectives reveal a distinct pattern, hinting at a more sparse and hierarchically organized wiring configuration compared to other scenarios.

The analysis results indicate a predisposition towards a higher ratio of backward paths compared to forward paths across the network. This inclination, coupled with the observed bias in connectivity between different subtypes, underscores the presence of intra-subtype connections that facilitate lateral information transfer. This aspect is particularly intriguing as it mirrors the biological significance of feedback mechanisms in higher-order processing and control within neural circuits. However, the extent to which these findings directly imply a similar functional role in the modeled networks remains a subject for further investigation.

### Network functionality analysis

As shown in Fig. 4, we conducted a wiring pattern analysis of patterns with random connections (${{\varvec{W}}}_{rand}$), the highest TE connections (${{\varvec{W}}}_{top}$), and the lowest TE connections (${{\varvec{W}}}_{bot}$). In this section, we analyze the information-processing characteristics of the generated network (hereafter referred to as the characteristic analysis).

**Figure 5:**
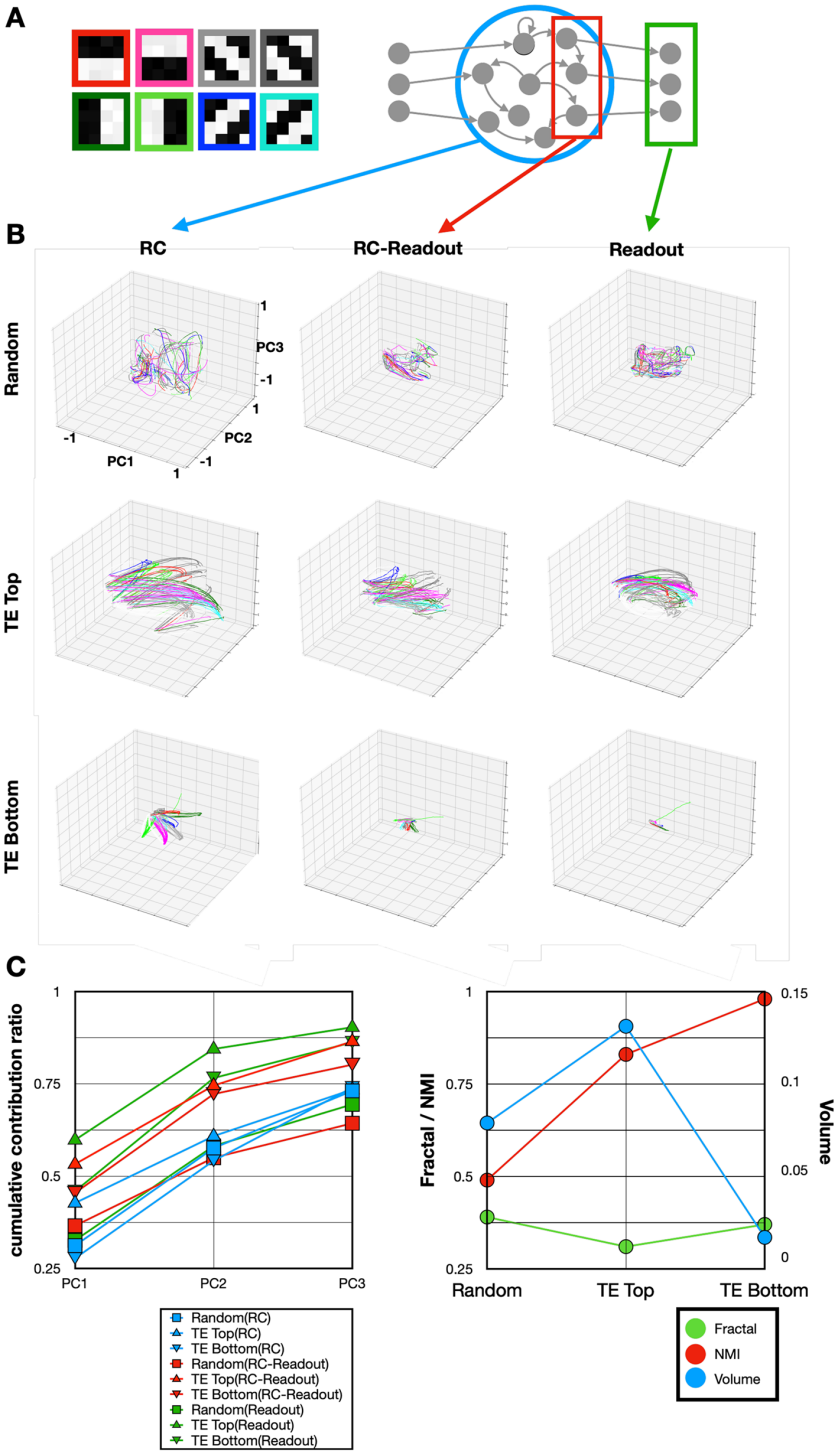
Functional characteristics analysis of the network. (**A**) Input stimuli utilized for the characteristic analysis are depicted on the left, and the regions where the analysis was conducted are shown on the right. The analysis focused on the dynamics within the reservoir layer (blue), RC-Readout units (red), and readout units (green). (**B**) 3D trajectory diagrams post-principal component analysis (PCA) of each dynamic, with line colors matching the stimulus frame colors from A. The layout illustrates the dynamics for randomly initialized entities, entities with the highest TE, and entities with the lowest TE, arranged from top to bottom. The sequence from left to right reflects the dynamics within the entire reservoir layer (blue), RC-Readout units (red), and readout units (green) for each type of entity. (**C**) (Left) Cumulative contribution rates diagram up to the third principal component for each entity type, with line colors indicating the analysis location. Markers (upper triangle for top entities, lower triangle for bottom entities, and square for random entities) distinguish between the different entity performances. (Right) Shows the evolution of the fractal dimension (green), normalized mutual information (NMI) (red), and trajectory volume (blue) across the patterns. The horizontal axis is divided, with the fractal dimension and NMI on the left, and trajectory volume on the right.

The characteristic analysis conducted on the network investigates the information-processing capabilities in response to a set of eight distinct stimuli, encompassing horizontal, vertical, diagonal downward, and diagonal upward orientations, as depicted in Fig. 5A. To mimic natural visual processing conditions, random noise was superimposed on each stimulus from a uniform distribution between [0, 0.1], and stimuli were presented as cropped images in a sequence, akin to the stimulus time-series generation flow outlined in Fig. 1C. Unlike the training and validation phases, where each stimulus was presented for a singular time step, this phase extended the duration of each stimulus presentation to n time steps to allow for a comprehensive dynamic analysis. An interval devoid of any stimulus for n time steps was also incorporated between successive stimulus presentations, facilitating the observation of network responses to the absence of visual input.

Figure 5B utilizes a 3 × 3 grid to compare the dynamics of ${{\varvec{W}}}_{rand}$, ${{\varvec{W}}}_{top}$, and ${{\varvec{W}}}_{bot}$ across rows against the reservoir layer, RC-readout unit, and readout unit dynamics, organized in columns. The color scheme within each plot corresponds to the stimulus types introduced in Fig. 5A, with the border colors around each stimulus image indicating the respective trajectory colors in Fig. 5B. The depiction of dynamics within each plot is achieved through dimensionality reduction, specifically principal component analysis (PCA), reducing the data to three dimensions based on the first three principal components. This method standardizes the dynamics across units, enabling a visual and quantitative assessment of how each network configuration processes and responds to a variety of visual stimuli.

The analysis yields several key insights: In the comparison within the leftmost column, ${{\varvec{W}}}_{rand}$ does not demonstrate selective responsiveness to stimuli within its internal dynamics, unlike ${{\varvec{W}}}_{top}$ and ${{\varvec{W}}}_{bot},$ which exhibit marked selective responsiveness. This distinction implies that the developed wiring patterns possess attributes akin to the filter circuits observed in the natural visual system, indicating the development of functionalities that mirror those found within biological visual systems^40,41^.

To assess the dynamics’ stability and separability, measures such as FD and NMI were employed. The FDs for ${{\varvec{W}}}_{top}$ and ${{\varvec{W}}}_{bot}$, at 0.31 and 0.37, respectively, show a slight difference, with ${{\varvec{W}}}_{top}$ exhibiting a lower value. This suggests that ${{\varvec{W}}}_{top}$ is characterized by lower complexity and enhanced stability (Fig. 5C, green). Separability metrics, with values of 0.83 for ${{\varvec{W}}}_{top}$ and 0.98 for ${{\varvec{W}}}_{bot}$, indicate a higher degree of class distinction in ${{\varvec{W}}}_{bot}$’s trajectories (Fig. 5C, right red).

In terms of the spatial volume of the trajectories, ${{\varvec{W}}}_{top}$ demonstrates the broadest trajectory volume among the configurations examined, with its dynamics occupying a space approximately six times larger than that of ${{\varvec{W}}}_{bot}$ (Fig. 5C, right blue). This finding underscores the necessity of expanding the dynamics' representational range within a constrained dimensional space to optimize information transmission.

Further examination of the dynamics across the network from left to right within each row reveals a trend of narrowing dynamic range. Given the uniform scale across the figures, it becomes apparent that despite ${{\varvec{W}}}_{bot}$’s internal selectivity, it falls short in effectively transmitting information to the RC-Readout and readout units. In contrast, ${{\varvec{W}}}_{rand}$ lacks consistent dynamics, highlighting its inefficiency in representing and conveying information. On the other hand, ${{\varvec{W}}}_{top}$ distinguishes itself with superior information transmission capabilities.

The analysis of the dynamics within the RC-Readout and readout units, as detailed in Fig. 5C (left), offers critical insights into the selective responsiveness to stimuli, the breadth of the representation space of dynamics, and the flexibility of the network’s information processing capabilities. By examining the cumulative contribution rates of the principal components in PCA, it becomes evident that the dynamics of both ${{\varvec{W}}}_{top}$ and ${{\varvec{W}}}_{bot}$ are primarily embedded in a low-dimensional space, as indicated by the significant contributions from the first to third principal components. This embedding suggests that information is efficiently encoded and transmitted downstream from the RC layer to the RC-Readout and readout layers, highlighting the network’s ability to embed information within a manageable dimensional scope.

Interestingly, even the dynamics of the readout connections for ${{\varvec{W}}}_{bot}$ (represented by green triangles in Fig. 5C) demonstrate a level of information embedding comparable to that of ${{\varvec{W}}}_{top}$. This finding implies that the process of information embedding and transmission from the input layer to the readout layer is executed effectively across both patterns. However, a critical distinction arises in the strength of transmission within the wiring pattern of ${{\varvec{W}}}_{top}$, which, despite appropriate information embedding, fails to achieve efficient information transmission, as reflected in the lower TE.

Further analysis across different conditions, as illustrated in Fig. 3, consistently shows ${{\varvec{W}}}_{top}$ exhibiting larger trajectory volumes than ${{\varvec{W}}}_{bot}$, with the FD revealing lower complexity and greater stability for ${{\varvec{W}}}_{top}$. However, the separation metrics across conditions do not consistently favor ${{\varvec{W}}}_{top}$ over ${{\varvec{W}}}_{bot}$. Notably, ${{\varvec{W}}}_{top}$ patterns with higher separation, particularly under constraints focused solely on connection costs (such as the left figure in the third row of Fig. 5B), indicate that while separation is critical, it does not directly equate to higher TE. A balance of separation and dynamic diversity appears to be optimal for effective information transmission.

The qualitative aspects of the analysis suggest that including a reduction in coupling costs as an objective enhances the separation of dynamics. Conversely, targeting an increase in TE as a goal tends to consolidate the dynamics into more elliptical trajectories while expanding the representation space. This dichotomy underscores the intricate balance between achieving separation in the dynamics for clarity in information processing and maintaining a sufficient diversity and range of dynamics to ensure robust and flexible information transmission.

## Discussion

This study explores circuit formation in the visual system by framing it as a multi-objective optimization problem. Utilizing a genetic algorithm, this study integrates objectives related to information transmission and network constraints, such as maintenance costs, to generate and analyze trends in wiring patterns and their information processing characteristics. Unlike many theoretical investigations focused on the visual system^42–44^, this study distinguishes itself by considering circuit evolution through the lens of multi-objective optimization, incorporating factors beyond network learning and postnatal visual experiences. This approach has unveiled the emergence of selectivity to stimuli—a hallmark of actual visual systems—based on the observed wiring patterns and their processing traits, demonstrating the study’s novelty.

We explored the relationship between changes in wiring patterns and their effects on TE, coupling costs, and density. We concluded that efficient information transfer needs sparse circuits with internal modular structures that use distinct wiring patterns. Wiring patterns that facilitate efficient information transfer exhibit filter-like behaviors akin to those observed in the dynamic activities within our visual system. The emergence of new functionality requires optimizing information transmission efficiency while minimizing maintenance costs.

Key findings from this research highlight the emergence of circuits with hierarchical organization and clear preservation under conditions that prioritize a reduction in network density. These outcomes underline the significance of density in the formation of wiring patterns, pointing towards the essential wiring configurations required for efficient information transmission. Despite these advancements, the study acknowledges the challenge of definitively identifying anatomically reported network structures, such as feedforward types, solely based on the results obtained. It also posits that patterns exhibiting high TE are not necessarily the optimal overall configurations. The variability observed under condition 5, which is aimed at optimizing all objective functions, suggests that achieving a balance among these objectives is pivotal for optimal network performance. Moreover, the effectiveness of sparse structures, as indicated by the results, resonates with the architectural principles observed in biological circuits, emphasizing the role of sparse connections in hierarchical feature representation, selectivity realization, and energy efficiency enhancement.

The complexity of actual biological tissues, characterized by the morphology of patterned neurons and the heterogeneity of cell distribution, hints at additional factors influencing circuit formation beyond those considered in this study^45–48^. Such complexities suggest that the conditions and objective functions defined here represent only a subset of the various influences shaping the acquisition of anatomically observed structures. The reduction of redundancy in information processing and the selective separation of trajectories, as observed in this study, align with characteristics reported in actual sensory systems^43,44,49–54^. These phenomena underscore the efficiency of neural circuits in filtering and representing information in a manner that maximizes transmission while minimizing unnecessary overlap, resonating with the findings of Bell and others who have emphasized the importance of circuit structures that bolster information transmission within the visual system. Furthermore, the ability to selectively respond to specific patterns, mirroring the response characteristics of the visual system, exemplifies the use of independent representations as a superior strategy for information transmission^55,56^. The findings of this study confirm these principles by demonstrating that selective responsiveness and the effective separation of trajectories contribute to the enhanced representation of information. However, the observation of clear trajectory separations in lower-performing patterns highlights a potential trade-off between the diversity of information representations and the clarity of signal transmission. To optimize information throughput, the study suggests that information should be efficiently transmitted, as illustrated in Fig. 5A (right), and mechanisms for conveying stimulus-specific information effectively must be employed.

The dynamics associated with the highest pattern, characterized by separation and flexibility, were deemed most effective in this context. The increase in TE and the expansion of dynamic trajectory volume indicate that reducing binding costs contributes to the diversity of information representations. This study’s insights reveal that a balance between moderate selectivity and diversity in response to stimuli, combined with a considerable trajectory volume and well-structured paths for information flow from input to output, constitutes an optimal configuration for enhancing information transmission.

In essence, this research highlights the intricate balance required in neural circuit design to achieve efficient information processing. By integrating moderate selectivity, diversity, and expansive trajectory volumes, neural networks can mirror the sophisticated processing capabilities of biological systems. This balance is crucial for developing neural network models that not only replicate but also extend the functionalities observed in natural sensory systems, offering profound implications for the fields of computational neuroscience and artificial intelligence.

The findings of this research underscore the notion that circuit structures are not solely shaped by postnatal learning but are also genetically encoded, reflecting optimal configurations honed through evolutionary processes^2,4,5,7,53^. This genetic determination suggests an inherent capacity for self-organization within neural circuits, leading to common patterns of response selectivity that have been preserved across species sharing similar survival strategies. Such self-organization underscores the fundamental role of evolutionary pressures in sculpting the neural architectures that underpin specific sensory and information processing capabilities.

The implications of these insights extend beyond the realms of biology and neuroscience, offering valuable perspectives for the advancement of computational models, particularly in the domain of RC. In RC systems, where the non-readout connections are fixed and non-plastic, the performance is inherently dependent on the initial internal structure. The challenge lies in devising a generalizable strategy for the initialization of these structures to optimize performance. The study’s findings advocate for the strategic organization of neural networks to facilitate the self-organized acquisition of modality-specific information processing functions.

By applying principles of self-organization and structural optimization, RC models can be tailored to maximize information transmission across various modalities, including visual and auditory inputs, without being constrained by specific task requirements or input types. This flexibility ensures that RC systems can adapt to a wide range of tasks while maintaining high performance levels. Furthermore, the selection of structural conditions allows for the imparting of dynamics characteristic of any desired property, opening up new avenues for customizing RC systems to exhibit desired characteristics. This enhances their applicability and effectiveness across various applications^8,21,22,57,58^.

In conclusion, this study not only enriches our understanding of the evolutionary and genetic underpinnings of neural circuit formation but also illuminates pathways for the innovative design and optimization of computational models like RC. By drawing parallels between the evolutionary optimization of biological circuits and the structured organization of RC systems, this research paves the way for the development of more efficient, flexible, and adaptable computational architectures, inspired by the intricate and evolved mechanisms of natural information processing systems.

### Supplementary Information

Supplementary Figures.

Supplementary Legends.

## Data Availability

The source code (Python code) is available at [10.5281/zenodo.10801126].
