# Supplementary figures and images for: Emergence of input selective recurrent dynamics via information transfer maximization
Source: Sci Rep. 2024 Jun 13;14:13631. doi: 10.1038/s41598-024-64417-6 (PMC11176313; doi:10.1038/s41598-024-64417-6)

**A**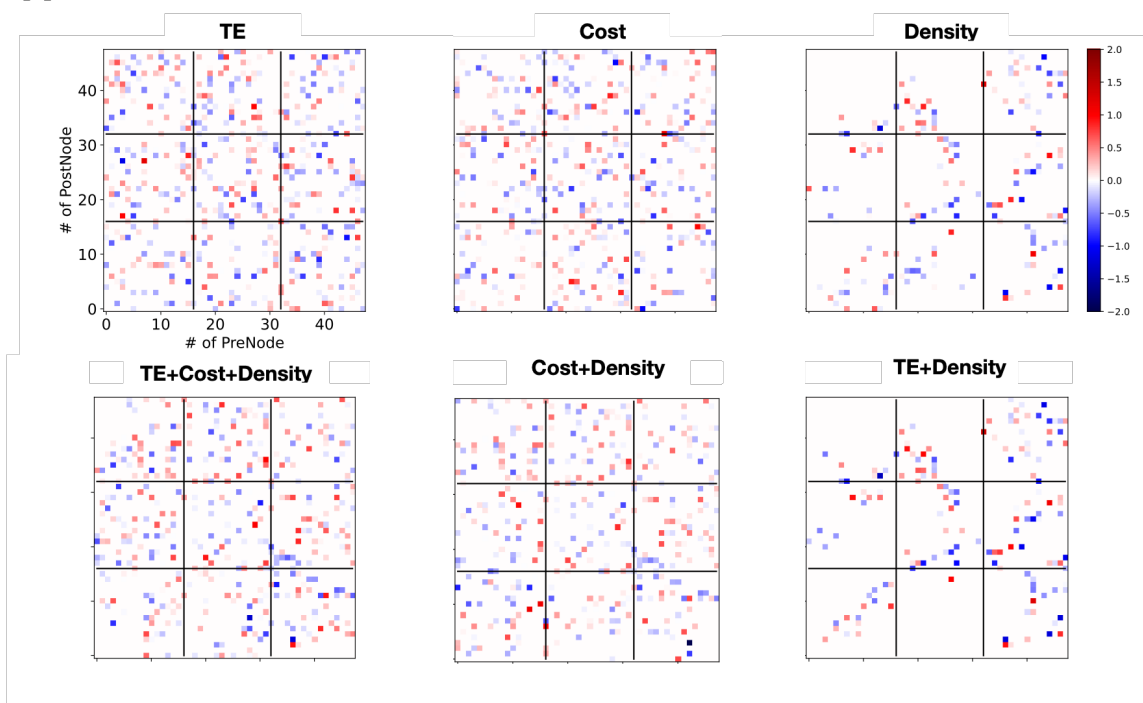**B**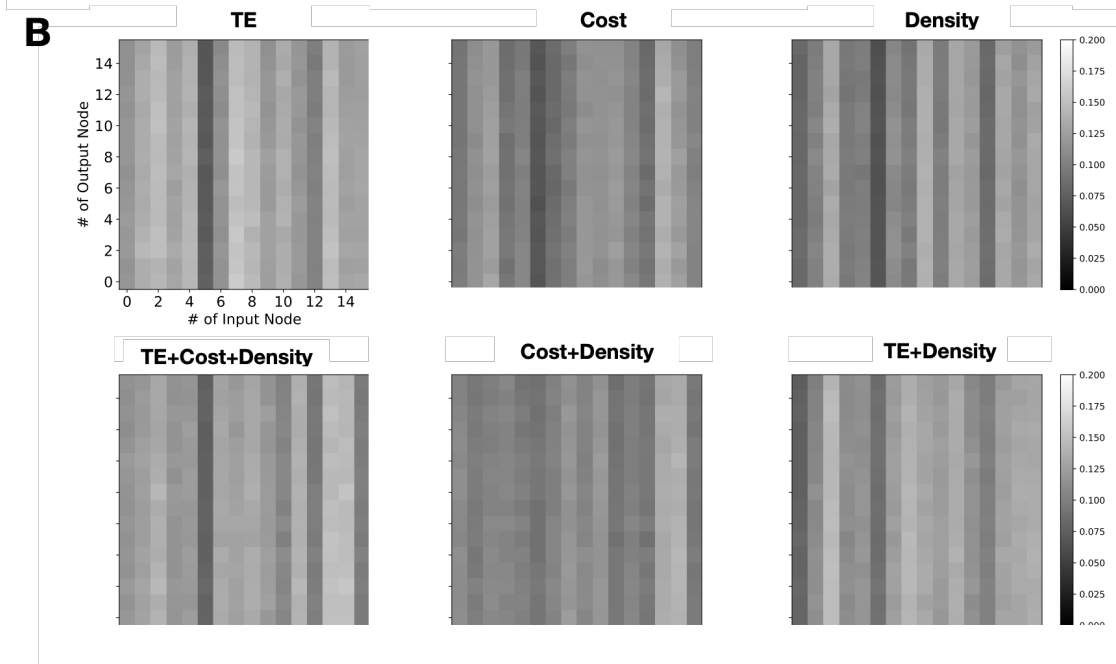**C**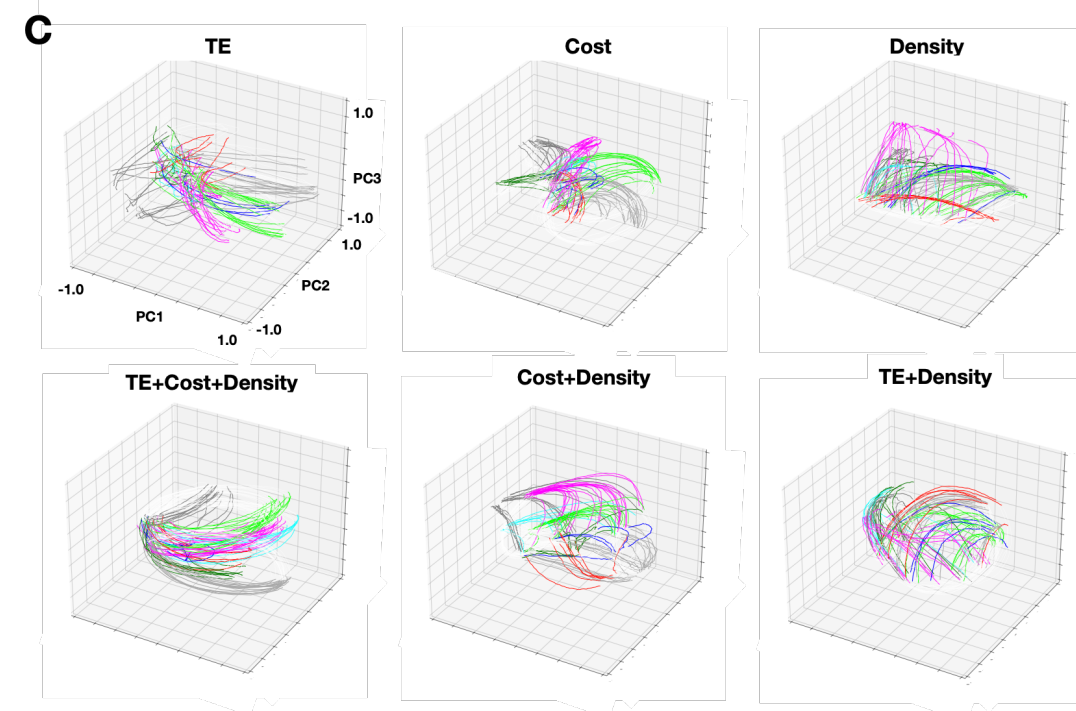**S Figure 1**

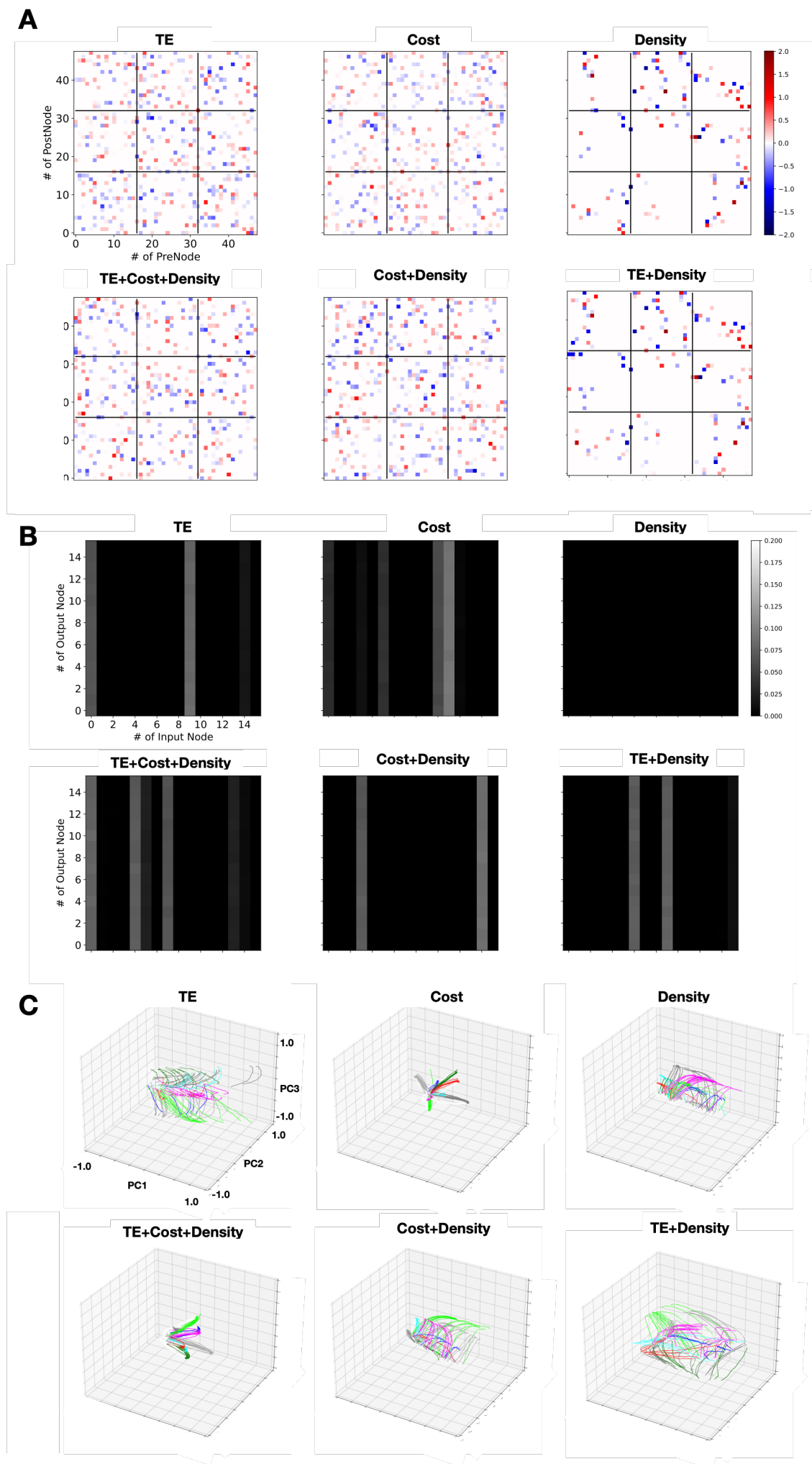

**S Figure 2**

Supplement: Supplementary file 1 — Supplementary Figures. [file 41598_2024_64417_MOESM1_ESM.pdf]
