# Supplementary material for: Emergence of input selective recurrent dynamics via information transfer maximization
Source: Sci Rep. 2024 Jun 13;14:13631. doi: 10.1038/s41598-024-64417-6 (PMC11176313; doi:10.1038/s41598-024-64417-6)
Supplement: Supplementary file 2 — Supplementary Legends. [file 41598_2024_64417_MOESM2_ESM.docx]

**Supplementary Materials**

**Sfig. 1. Analysis of Wiring Patterns and Characteristics for Top TE Patterns Across Various Conditions**

A: Coupling patterns for the top TE patterns as detailed in Fig. 3 for each experimental condition.

B: Amounts of TE transferred for the top TE patterns under each experimental condition presented in Fig. 3.

C: Dynamic trajectories within the reservoir layer. The upper row delineates structural trends for specific conditions: solely increasing TE, solely decreasing coupling cost, and solely decreasing density (left to right). The lower row outlines structural trends for combined conditions: increasing TE alongside coupling cost reduction, increasing TE with density reduction, and coupling cost reduction combined with density reduction (left to right). Line colors in C match the stimulus frame colors shown in the right panel of Fig. 5A.

**Sfig. 2. Analysis of Wiring Patterns and Characteristics for Bottom TE Patterns Across Various Conditions**

A: Coupling patterns for the bottom TE patterns across each condition detailed in Fig. 3.

B: TE transfer amounts for the bottom TE patterns under each condition outlined in Fig. 3.

C: Trajectories of dynamics within the reservoir layer. Each row highlights the structural trends under distinct conditions: increasing TE only, decreasing coupling cost only, and decreasing density only (from left to right in the upper row). The lower row shows the structural trends for conditions combining objectives: increasing TE with coupling cost reduction, increasing TE with density reduction, and coupling cost reduction with density reduction (from left to right). The line colors in C are consistent with the frame colors indicated in the right panel of Fig. 5A.

**
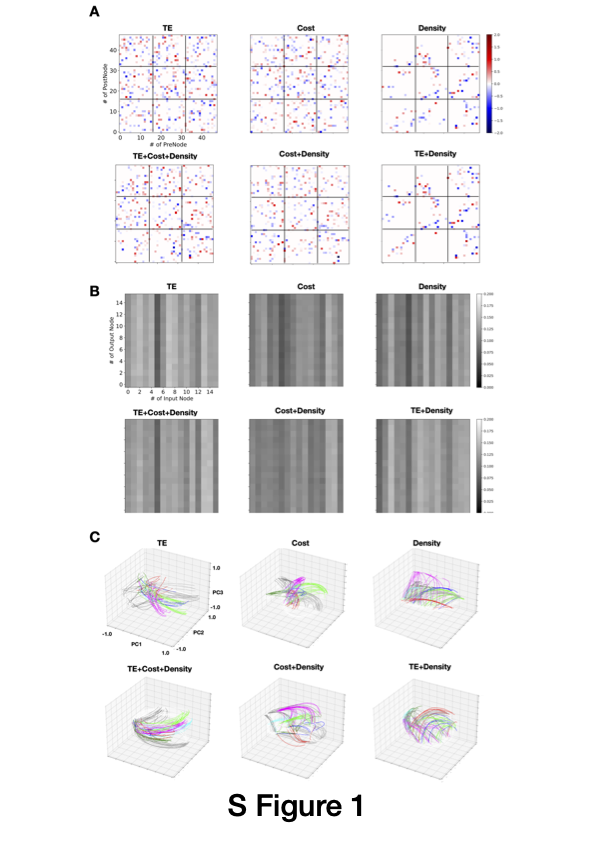
**

**
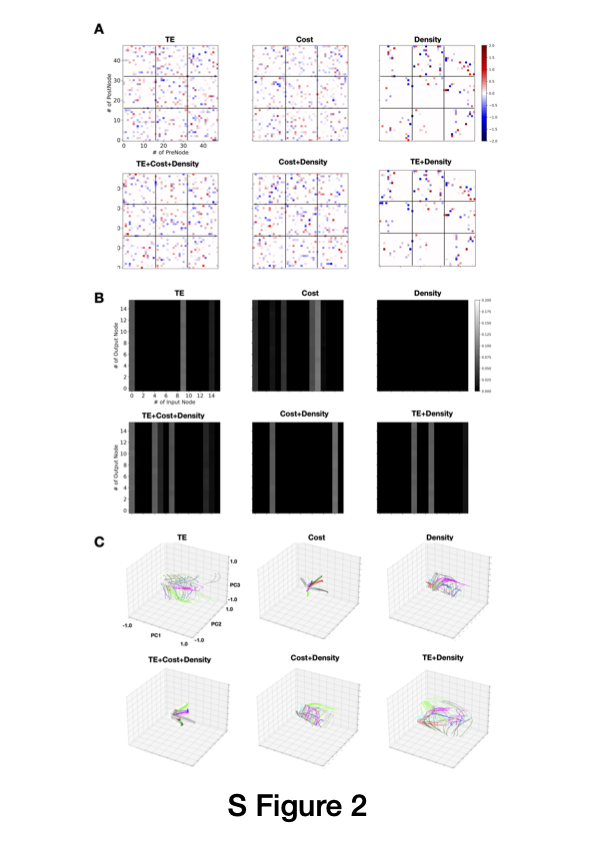
**
